# Assessing the Development of Collective Impact Initiatives Addressing Maternal and Child Health

**DOI:** 10.1007/s10995-020-02894-7

**Published:** 2020-02-12

**Authors:** Sarah Landry, Vicki Collie-Akers, Kara Foster, Denise Pecha, Chad Abresch

**Affiliations:** 1 Department of Population Health, University of Kansas School of Medicine, 3901 Rainbow Boulevard, Kansas City, KS 66160 USA; 2 University of Nebraska Medical Center, 982170 Nebraska Medical Center, Omaha, NE 68198-2170 USA

**Keywords:** Collective impact, Maternal and child health, Evaluation, Technical assistance

## Abstract

**Purpose:**

To examine the extent to which communities participating in the Collective Impact Learning Collaborative (CILC) increased capacity to create conditions for collective impact (CI) to address racial disparities in maternal and child health (MCH) and align local efforts with state MCH priorities over a 12-month period.

**Description:**

Eight communities participated in a learning collaborative that involved the provision of technical assistance via webinars, monthly team calls, and site visits to facilitate the development of a collective impact initiative. A Ready-Set-Go approach to technical assistance was used to guide the communities through each phase of development while also providing individual assistance to teams based on their capacity at the start of participation.

**Assessment:**

A pre/post design measured change in capacity to engage in CI efforts over time. A survey designed to assess the completion of core tasks related to early indicators of CI was completed at baseline and 12 months later. Wilcoxon Signed Ranks Test and Mann–Whitney test determined statistically significant progress towards outcomes over 12 months and differences in progress between high- and low- capacity teams.

**Conclusion:**

In 12 months, teams with little established groundwork made significant progress, in some ways exceeding progress of more established teams. Statistically significant progress was achieved in eleven of fourteen outcomes measured. Five teams aligned local efforts with state priorities after 12 months. Findings suggest technical assistance to establish conditions for collective impact can support progress even when pre-conditions for collective impact are not previously established.

## Significance

The life course perspective is often applied in practice and research related to racial and health disparities in MCH. This calls for novel approaches to collaboration in order to more fully integrate systems along the continuum of care that intervenes at various life stages.

Examination of the implementation of the first phases of a collective impact model suggest that a significant amount of progress can be made in a relatively short amount of time to establish the groundwork for collaboration and implementation of strategies to address racial disparities in maternal and child health and align local and state priorities regardless of capacity to begin the work.

## Purpose

Life course theory provides a framework to view stages of development (e.g. infancy, adolescence, adulthood) as an integrated whole where intervention to improve protective factors for health should occur at each phase to increase odds of better health outcomes at later phases (Fine et al. 2009). This approach requires public health organizations play a role in improving maternal and child health (MCH) clinical outcomes, but also early education, housing, community networks, and the private sector as they relate to influencing the quality of a school, safety of a home, connectedness to neighbors, and opportunity for employment across the span of one’s life. To improve MCH across the life course, diverse multi-sector collaboration is required (Lu 2014). Models of collaboration describe processes and principles that coalitions adopt when convening stakeholders to address complex issues (e.g. Butterfoss and Kegler 2009; Fawcett et al. 2010; Wolff 2010; Kania and Kramer 2011). However, a challenge posed by life course theory, is to move beyond collaboration to “multi-dimensional systems integration” (Lu 2014, p. 341). This approach means moving from a focus on impact of some organizations to developing capacity for collective impact across levels of intervention (e.g. individual, community), systems and sectors, and longitudinally across the continuum of care that intervenes at various life stages (Lu 2014).

The collective impact (CI) model is a useful framework for systems integration to address MCH issues from a life course perspective. Core components of the CI model include principles of collaboration, coalition development, and community organizing described in literature (e.g. Butterfoss and Kegler 2009; Fawcett et al. 2010; Wolff 2010). However, a focus of the CI model is aligning goals and activities across systems and sectors with support from a system of governance that promotes strategic planning, community involvement, and continuous evaluation (Preskill et al. 2014; Hanleybrown et al. 2012).

This paper explores the extent to which communities created conditions for CI to address MCH outcomes through technical assistance (TA) as a participant in the Collective Impact Learning Collaborative (CILC). The Collaborative was funded through the Health Resources and Services Administration’s (HRSA) Maternal and Child Health Bureau to create greater alignment between state and local MCH priorities. Federal funding for MCH is provided through Title V of the Social Security Act. Title V is a federal-state partnership providing funding and support to address complex problems like access to quality health care and reduction of infant mortality. The flexibility of the block grant allows state health departments (HD) to prioritize and fund the greatest needs.

While Title V is a federal-state partnership, many important elements of MCH programming are planned and implemented by local HDs. Local HDs may align priorities and activities to enhance progress toward the MCH needs identified by states. Thus, the CI model was used to focus on systems integration and alignment to support effective use of resources to address MCH outcomes. Establishing more evidence regarding the extent to which CI is a useful model for state and local alignment and what role technical assistance and community capacity might play in that process can inform best practice for community based MCH initiatives.

The CI model describes three pre-conditions and five phases of implementation. Pre-conditions include the presence of an influential champion, adequate financial resources, and a sense of urgency for change among community and partners (Hanleybrown et al. 2012). Five phases of CI include assessing readiness, initiating action, organizing for impact, beginning implementation, and sustaining action and impact (Preskill et al. 2014). During each phase, communities develop five conditions for CI: forming a common agenda, developing a shared measurement system, establishing mutually reinforcing activities, continuous communication, and creating a backbone infrastructure (Preskill et al. 2014).

## Description

CityMatCH, the National Organization of MCH leaders, entered a cooperative agreement with HRSA to provide technical assistance to local HDs to address Title V MCH priorities in their communities. HDs completed a questionnaire to assess readiness to begin a CI initiative. Seventy-one HDs completed the questionnaire. Thirty local HDs expressed interest in participation in the collaborative and had not received any previous training or TA support for CI. These HDs were invited to participate in one of the three cohorts beginning in 2016 through 2018. Each cohort consisted of representatives from ten local HDs and their community partners.

HDs were asked to assemble community representatives and HD staff to act as a core team responsible for mobilizing and organizing partners to address MCH priorities. Core teams varied in readiness to participate. Higher capacity teams met some pre-conditions for CI including having dedicated resources for a CI initiative, previously formed coalition, and knowledge about core principles of CI. Lower capacity teams had no financial resources or experience with the CI model, but in some cases had champions able to bring organizations together. Cohorts were arranged so both high- and low-capacity teams were included in each cohort to support peer mentorship. Analysis and results that follow include data from the first cohort of teams.

**Fig. 1:**
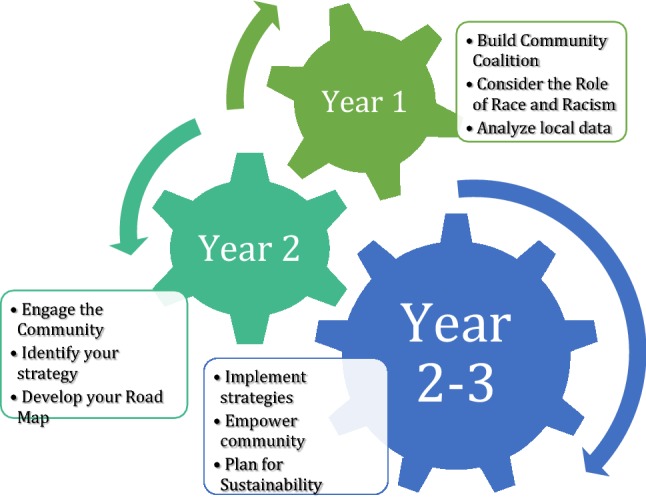
Ready-set-go framework

CityMatCH provided TA via webinars, monthly calls, and site visits based on a Ready-Set-Go framework (CityMatCH 2018). The Ready phase supported teams in building leadership, community and stakeholder buy-in, and gathering and analyzing information about priority MCH issues. Webinars with expert practitioners and monthly TA calls covered topics like connecting teams with their Title V Directors, core principles and strategies for CI, engaging community members and champions, and building a coalition with stakeholders from multiple sectors. The Set phase focused on narrowing down the work, prioritizing equity in CI work, and creating agendas for coalition meetings (e.g. tools, logistics, language and messaging). TA during the Go phase centered around evaluation and developing conditions for sustainability. However, this phase of TA was not implemented during the survey period reported. By providing TA, it was expected teams could engage in processes for spurring collaborative action and establish early conditions for CI that would address MCH priorities (Fig. 1).

## Assessment

A pre/post evaluation design measured change in team capacity to engage in CI efforts over time. A survey developed from the Best Change Processes framework was used (Roussos and Fawcett 2000). Completion of core tasks related to Best Change Processes increase a group’s ability to affect change, are associated with more robust community-level interventions (Keene-Woods 2014; Watson-Thompson 2015; Fawcett et al. 2010), and align with early outcomes for CI identified by Preskill et al. (2014).

Core tasks were indicators of progress as they related to specific outcomes proposed by Preskill et al. (2014) in their *Guide to Evaluating Collective Impact*. The number of completed tasks measured the extent to which communities produced early outcomes of success. Team leaders completed the survey prior to participation in the CILC and 12 months later. The survey consisted of 92 questions with “Yes”, “No”, or “Don’t Know” response options depending on completion of core tasks. One survey was completed per team. Respondents were asked to choose “Yes” if the task was fully completed, “No” if only partially completed, or “Don’t Know” if they were unsure. Evaluation protocols were submitted to University of Kansas IRB for approval and were deemed exempt from review.

Table 1 provides a list of early outcomes and examples of selected indicators.

**Table 1:** Phase 1 and 2 outcomes for success in collective impact

Core component of CI	Early outcomes	Example select indicators
Common agenda	Common agenda was developed that included a diverse set of voices and multiple sectors	Populations affected by the problem were identified and engaged in assessing its importance
	Partners achieved a common understanding of the problem	The group identified the type(s) of problems or goals that it will address to help focus its efforts
	Partners have consensus on the initiative’s ultimate goal and shared vision for change	The group has engaged stakeholders in the develop of a mission statement
	Partners are committed to using an adaptive approach with agreed upon strategies and actions	The group has written strategies that are approved by the group and state how goals will be accomplished
Backbone infrastructure	Backbone infrastructure effectively guides the CI initiative’s vision and strategy	Leaders guide important processes and act as facilitators
	Backbone infrastructure ensures alignment of existing activities and pursuit of new opportunities toward the initiative’s goal	The group reviews the strategic/action plan and makes necessary modifications and updates
	Backbone infrastructure supports the collection and use of data to promote accountability, learning, and improvement	The group regularly reviews the date about the implementation of the intervention or initiative
Mutually reinforcing activities	Partners developed and use a collective plan of action	Action plans indicate what will be done, by whom and by when
	Partners coordinate activities to align with plan of action	The group has written objectives to guide the efforts of the initiative
	Partners have (re)allocated resources in support of the collective impact initiative	The group prioritized the choice of strategies based on the importance of meeting obj. and feasibility of strategies
Shared measurement	Partners understand the value of the shared measurement system	The group identified the types of data and information important to stakeholders and other audiences
	The process of designing and managing the shared measurement system is participatory and transparent	The group developed a set of evaluation questions that are important to stakeholders and key audience
	The shared measurement system has been designed to track progress toward CI outcomes	The group documents the activities used to address the problem/goal
Continuous communication	Structures and processes are in place to engage partners, keeping them informed and inspired	The organization appropriately documents and record organizational activities (e.g. meeting minutes)
	Structures and processes are in place to engage the initiative’s external stakeholders, keeping them informed and inspired	The group regularly communicates progress toward completing the strategic/action plan to the staff, board, and other key stakeholders

The percent of tasks completed were calculated at baseline and 12 months. The number of “Yes” responses affirming completion of tasks were summed for each early outcome. The sum was then divided by the total number of possible tasks for each outcome measure to establish a percent complete in each community. The percent of completed tasks was averaged across communities to assess progress overall. This analysis was completed for high capacity and low capacity teams to compare progress between groups. The non-parametric Wilcoxon Signed Ranks Test determined if there were statistically significant differences within teams over time. The non-parametric Mann–Whitney test determined statistically significant differences between high- and low- capacity teams at baseline and 12 months. Two teams withdrew participation after several months due to staff turnover. Results include the eight remaining teams.

## Conclusion

Statistically significant progress was determined in eleven of fifteen outcomes (Table 2).

**Table 2:** Early outcomes of success for collective impact and community and system change

Early outcomes	Baseline	12 Months	
Common agenda	Average percent of completed tasks for achieving benchmarks (Mdn, IQR)	Average percent of completed tasks for achieving benchmarks (Mdn, IQR)	Wilcoxon signed-rank test Z score (p-value)
Common agenda was developed that included a diverse set of voices and multiple sectors	28% (37.5, 50)	69% (75, 18.75)	− 2.232 (.026)*
Partners achieved a common understanding of the problem	60% (50, 73)	87% (87.5, 25)	− 2.032 (.042)*
Partners have consensus on the initiative’s ultimate goal and shared vision for change	15% (7, 5.5)	56% (71, 50.75)	− 2.371 (.018)*
Partners are committed to using an adaptive approach with agreed upon strategies and actions	39% (36, 61.5)	67% (68, 28.5)	− 2.207(.027)*
Backbone infrastructure			
Backbone infrastructure effectively guides the CI initiative’s vision and strategy	19% (6.5, 34)	53% (56.5, 46.75)	− 1.970 (.049)*
Backbone infrastructure ensures alignment of existing activities and pursuit of new opportunities toward the initiative’s goal	29% (0, 67)	58% (66.5, 34)	− 1.372 (.170)
Backbone infrastructure supports the collection and use of data to promote accountability, learning, and improvement	12% (0, 9.75)	24% (19, 34.75)	− 1.581 (.114)
Mutually reinforcing activities			
Partners developed and use a collective plan of action	19% (0, 37.5)	25% (0, 75)	− 1.000 (.317)
Partners coordinate activities to align with plan of action	19% (8.5, 33)	63% (67, 41.5)	− 2.375 (.018)*
Partners has (re)allocated resources in support of the collective impact initiative	8% (0, 0)	33% (16.5, 83.3)	− 1.890 (.059)
Shared measurement			
Partners understand the value of the shared measurement system	17% (16.5, 33)	54% (67, 34)	− 2.226 (.026)*
The process of designing and managing the shared measurement system is participatory and transparent	6% (0, 0)	38% (50, 37.5)	− 2.236 (.025)*
The shared measurement system has been designed to track progress toward the CI’s outcomes	14% (0, 18.75)	41% (37.5, 68.75)	− 2.020 (.043)*
Continuous communication			
Structures and processes are in place to engage partners, keeping them informed and inspired	25% (0, 55)	73% (70, 35)	− 2.036 (.042)*
Structures and processes are in place to engage the initiative’s external stakeholders, keeping them informed and inspired	16% (0, 37.5)	69% (75, 50)	− 2.414 (.016)*

*p < .05

Teams made most progress ensuring inclusion of diverse sectors in developing leadership and a common agenda. Core tasks included establishing leadership inclusive of diverse skill sets, experience, interest/expertise, and ensuring community stakeholders had an opportunity to analyze information about the issue and assess its importance. Two teams indicated development of a common agenda at baseline. Neither of the teams with common agendas at baseline demonstrated state alignment. Alternatively, five teams that developed common agendas during the first 12 months of the CILC were aligned with state priorities. Teams made considerable progress towards establishing processes to engage external stakeholders by providing opportunities to propose strategies and solutions and regularly communicate progress towards developing and completing an action plan. Progress made in these areas is not surprising as they are natural places to begin collaborative work and are required to establish support and a common understanding of the problem and solution. Less progress was made toward establishing a backbone infrastructure, mutually reinforcing activities, and a shared measurement system. These conditions are more challenging to address and are often dependent upon successes related to developing a common agenda, leadership, and building trust among stakeholders. For example, progress toward developing mutually reinforcing activities and shared measurement may be hindered if groups are unable to fully form a common agenda. Similarly, identifying leadership and building trust within the community and among new partners is required to make progress establishing a backbone infrastructure where one doesn’t previously exist.

**Fig. 2:**
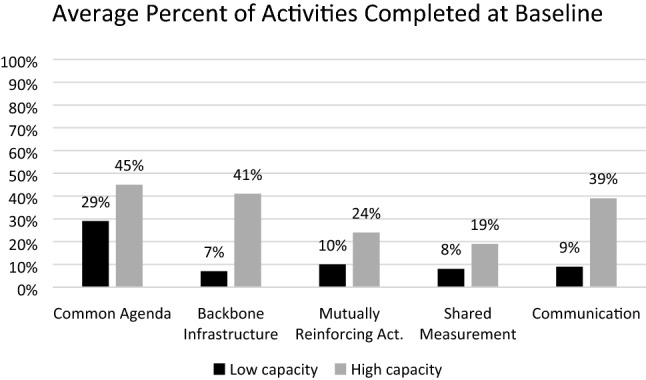
Average percent of activities completed at baseline

**Fig. 3:**
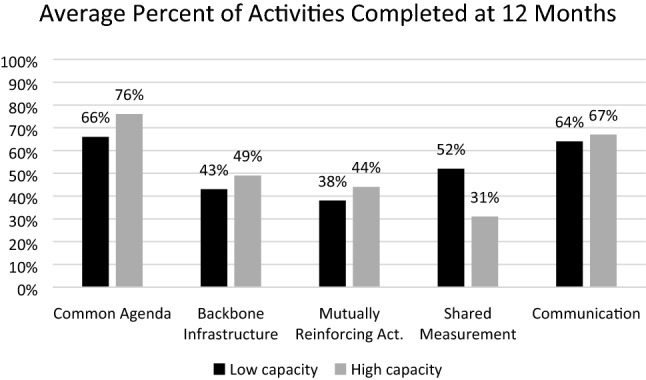
Average percent of activities completed at 12 months

Statistically significant differences were not identified at baseline or 12 months between high- and low-capacity teams. However, teams with lower levels of capacity often exceeded the progress of high capacity teams. Figures 2 and 3 display the average percent of tasks completed at baseline and 12 months by low (n = 5) and high (n = 3) capacity teams.

### Common Agenda

Prior to participating, low- and high-capacity teams took similar steps to lay the groundwork to develop a common agenda. For example, all teams had identified the types of problems or goals they hoped to address. Similarly, all but one team had identified where or among whom their efforts should be focused. However, teams with higher capacity had analyzed data, identified risk and protective factors, established vision and mission statements, and identified potential strategies for addressing issues prior to participation.

Teams with lower capacity made substantial progress collecting and reviewing data about the issues and getting input from community. These teams identified behavioral and environmental factors related to their priorities, established vision and mission statements, and reviewed evidence-based strategies to address the issue. Higher capacity teams completed these steps prior to baseline and could focus attention on aligning goals and strategies among stakeholders.

### Backbone Infrastructure

High capacity teams had taken several steps toward establishing a backbone infrastructure at baseline. Teams had a clear governing structure, method for operating (e.g. committees, staffing), and leadership able to connect the group to appropriate resources. None of the lower capacity teams reported these accomplishments at baseline. However, within 12 months, three lower capacity teams reported reaching those milestones. Considering few teams entered the CILC with backbone organizations established, it is to be expected that most of the focus during this period was on developing the infrastructure itself.

### Mutually Reinforcing Activities

The amount of action taken to establish mutually reinforcing activities were similar at baseline and at 12 months, regardless of team capacity. After 12 months, one high and one low capacity team, completed an action plan and prioritized strategies to implement. By 12 months, all lower capacity teams brought stakeholders together to develop a strategic plan including components like a clear mission and objectives.

### Shared Measurement

Although one high capacity team established a shared measurement system prior to participation, all other teams had little established at baseline. After 12 months, all teams in the low capacity group identified the type of data important to stakeholders. Similarly, four low capacity teams identified data sources and assured access to measures. Regardless of capacity, four of the eight teams made considerable progress towards designing a system to track outcomes, especially to document activities and collect longer-term measures. Unlike other components of CI, lower capacity teams made more progress in this area.

### Communication

Low capacity teams outperformed high capacity teams in establishing structures and processes for continuous communication. Two of the three high capacity teams had established processes prior to the start of the CILC leaving less room for progress. However, all low capacity teams began participation with either new or yet-to-be developed groups of stakeholders. These teams viewed the establishment of roles and responsibilities, meeting schedules, and development of other methods for communication as priority for developing a common agenda and mutually re-enforcing activities.

## Discussion

Overall most progress was made toward developing a common agenda and establishing communication mechanisms among partners. Taking action in these areas are the easiest and most logical first steps towards creating conditions for collective impact. Future assessment may permit the establishment of patterns of activity over time.

Teams made similar progress regardless of level of capacity at baseline indicating that pre-conditions are important but should not preclude teams from engaging in CI. Teams with little established groundwork made an exceptional amount of progress and, in some ways, exceeded progress made by more established teams. These findings may have a few explanations. Lower capacity teams developed new partnerships to support the work which appeared more open to fully adopting a CI framework. Core teams reported that TA provided the accountability needed to bring new partners along and accelerate progress during the initial phases of the work. High capacity teams worked within long-standing coalitions which offered benefits and challenges. Coalitions were large in number and able to generate resources and support at high levels (e.g. State HDs). However, core teams often found it harder to garner support of decision-makers to move away from the status quo and towards a new way of operating, particularly when establishing shared measurement systems. Established infrastructure and support for CI can be advantageous to bring communities closer to acting on MCH priorities within 12 months. However, it may come with challenges that slow progress by requiring core team members to put more effort on garnering support for new approaches to the shared work.

Core teams may have made progress in these areas without the direct involvement of TA support. However, a common refrain among all core teams was that TA provided knowledge, encouragement, and accountability which furthered progress beyond what they may have achieved on their own. TA providers helped teams reflect on progress by asking hard questions to move out of comfort zones, focus on core principles of CI, and provide benchmarks and timelines for progress. Communities interested in organizing to align activities and resources to address MCH outcomes can benefit from technical assistance that combines expert sources of information, encouragement, and accountability coupled with a clear CI framework for action regardless of pre-existing infrastructures or resources to begin the work.
